# Polymeric Insulator Conditions Estimation by Using Leakage Current Characteristics Based on Simulation and Experimental Investigation

**DOI:** 10.3390/polym14040737

**Published:** 2022-02-14

**Authors:** Ali Ahmed Salem, Kwan Yiew Lau, Zulkurnain Abdul-Malek, Nabil Mohammed, Abdullah M. Al-Shaalan, Abdullrahman A. Al-Shamma’a, Hassan M. H. Farh

**Affiliations:** 1 Institute of High Voltage and High Current, School of Electrical Engineering, Universiti Teknologi Malaysia, Johor Bahru 81310, Malaysia; zulkurnain@utm.my; 2 Department of Electrical and Computer Systems Engineering, Monash University, Wellington Rd., Clayton, VIC 3800, Australia; nabil.mohammed@ieee.org; 3 Electrical Engineering Department, College of Engineering, King Saud University, Riyadh 11421, Saudi Arabia; shaalan@ksu.edu.sa (A.M.A.-S.); ashammaa@ksu.edu.sa (A.A.A.-S.); 4 Department of Building and Real Estate, Faculty of Construction and Environment, Hong Kong Polytechnic University, Hung Hom, Kowloon 999077, Hong Kong; hfarh.hussein@polyu.edu.hk

**Keywords:** polymer insulator, prediction, pollution, leakage current indices

## Abstract

The current work contributes an estimate of the time-frequency characteristics of a leakage current in assessing the health condition of a polluted polymeric insulator. A 33 kV polymer insulator string was subjected to a series of laboratory tests under a range of environmental conditions, including pollution, wetting rate (WR), non-soluble deposit density (NSDD), and non-uniform distribution pollution (F_T/B_). The temporal and frequency features of the leakage current were then extracted and used as assessment indicators for insulator conditions based on laboratory test findings. Two indices were generated from the leakage current waveform in the time domain: the curve slope index (F_1_), which is determined by measuring the inclination of the curve between two successive time peaks of the leakage current, and the crest factor indicator (F_2_). The frequency domain of the leakage current signal was used to calculate the other two indices. These are the odd harmonic indicators derived from the odd frequency harmonics of the leakage current up to the 9th component (F_3_) and the 5th to 3rd harmonics ratio (F_4_). The findings showed that the suggested indicators were capable of evaluating insulator conditions. Finally, the confusion matrix for the experimental and prediction results obtained with the proposed indices was used to assess which indicator performed the best. Therefore, the analysis suggests an alternative and effective method for estimating the health condition of a polluted insulator through leakage current characteristics obtained in the time and frequency domains.

## 1. Introduction

The insulator is one of the most significant components used in power transmission systems for holding and insulating electrical wires from towers. In this respect, it should be noted that several environmental elements, such as the kind of material, moisture, and the level of contaminants, have a significant impact on the efficacy of the outdoor insulator. Consequently, the continuation of pollutants deposited on the surface of insulators renders the insulators susceptible to significant leakage current flow. As a result, the insulator surface experiences widespread discharge activity [1,2,3,4]. These discharges can develop into an unwanted flashover that may disrupt an electrical grid [5,6,7,8]. Therefore, monitoring the condition of insulators has a significant effect on the power system stability [9,10]. The assessment of outdoor insulator properties and long-term efficiency is thus an important area of research in the science establishment [11,12,13,14].

The leakage current (LC) monitoring method has several advantages. It considers a variety of environmental factors such as ambient temperature, moisture, contamination, and rain [15]. Additionally, the leakage current may simply be checked online regularly. Leakage current computation and analysis, as one of the online tests performed for contaminated insulators, has piqued the interest of numerous researchers. The author of [16] has utilized a microwave-disapproved device to measure the LC on dry insulator surfaces. However, this technology is expensive and may be too expensive for low-cost power systems. The authors of [17] proposed a device that captures electromagnetic radiation from a partial discharge to monitor the leakage current of contaminated insulators. Although this system is not affected by flashover, it has not yet been tested in the field, where considerable electromagnetic interference caused by coronae and other effects on high voltage cables is expected. Moreover, another interesting aspect of the contaminated insulator LC tracking approach is the ability to create an effective correlation between the leakage current and the insulator status when the insulator is in operation. In this scenario, several academics have offered different approaches to assess the state of insulators [15,18,19,20]. By extracting information on the components of the LC, it is possible to improve LC-based monitoring. The authors of [21] proposed measuring the degree of contamination using LC summary statistics, which offer the mean, peak, and standard deviation values. They made it clear that these factors allow them to measure the size and density of contamination on the insulator surface. Another study [19] calculated insulator pollution conditions by monitoring the shift angle between the leakage current and applied voltage signals. As per the results in [19], shift angle alterations are a helpful signal for assessing contaminants and moisture variations between clean and dry settings. Other strategies may be used to anticipate insulators’ pollutant incidence. This is only one of several approaches that have been developed in the field of insulators. Aside from that, a method known as leakage current component extraction, which has been used by various other studies [22,23,24], is a popular methodology that may assist in forecasting the pollution incidence of an insulator. In this procedure, the Fast Fourier Transform (FFT) and wavelet transform are employed to examine the leakage current signal in the frequency domain. Overall, the findings demonstrate that contamination on the insulator increases leakage current harmonic components, particularly the odd harmonics. Furthermore, the results indicate that pollution increases the 1st and 3rd harmonics as well as the total harmonic distortion (THD) [25]. According to the results in [26], the harmonics in question are the first and third components of an AC system with a frequency of 50 Hz. Consequently, the study found that increasing these harmonics causes an increase in the total harmonic distortion (THD), which varies depending on the degree of pollution and applied voltage harmonics.

Recently, the pre-flashover circumstances of polymeric insulators under polluted environments were explored by utilizing a mix of analytical formulations based on the hybrid genetic algorithm and particle swarm optimization (HGA-PSO) method [26]. The results demonstrated that, under various pollution and pre-flashover scenarios, the relationship between the discharge resistance and the leakage current of experimental specimens were similar to those obtained through analytical formulations in the literature. There was a close connection between genuine experimental data, circuit analytical modeling from prior studies, and the HGA-PSO system. In [27], the time-frequency domain surface leakage current (SLC) signals of an 11 kV polymeric insulator with a polluted surface were analyzed through hyperbolic window Stockwell transform (HST). The authors of [27] concluded that the suggested HST-based feature extraction approach could be used for polymeric insulator status monitoring.

It is highly advantageous to have an indicator that reflects the status of the insulators, as proposed in [15,19]. The authors of [28] used the basic assumption of the frequency components of the leakage current to construct the related indices throughout the context of this issue. When calculating flashover accidents, the indicator which determines the ratio of the 3rd to 5th harmonics of the leakage current and THD was used. The reported results for glass and polymeric insulators revealed a strong relationship between the extent of contamination and the reading of this indicator. Nonetheless, a literature survey revealed that no attempt had been made to compare the conditions of the insulators using different indices that take into account the time signal slope and frequency harmonics up to the 9th component for the leakage current. Compared to the leakage current indicator (3rd/5th) proposed in [28], this approach is expected to produce a more dependable estimate.

The current work aimed to estimate the polymeric insulator condition under different environmental circumstances based on leakage current characteristics in the time and frequency domains extracted experimentally. To simulate the natural medium of insulators during service, the environmental parameters pollution level (SDD), wetting rate (WR), non-soluble deposit density (NSDD), and the non-uniform distribution of pollution (F_T/B_) were taken into account. The results of the proposed leakage current indicators under the influence of environmental conditions were extracted experimentally. To assess the performance of these indicators, the confusion matrix for experimental data and prediction results using the suggested indices was employed.

## 2. Materials and Methods

### 2.1. Test Sample

**Figure 1:**
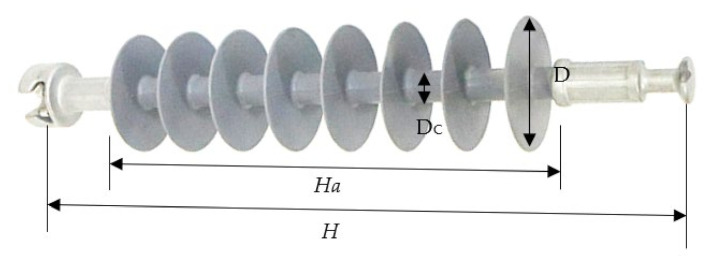
33 kV polymer insulator sample.

The investigated polymer insulators were obtained from the “Transmission Division of Malaysian National Power (TNB)” in order to conduct the experiment. Figure 1 illustrates the main shape of the chosen insulator. Table 1 details the insulator’s characteristics.

### 2.2. Experimental Setup

**Figure 2:**
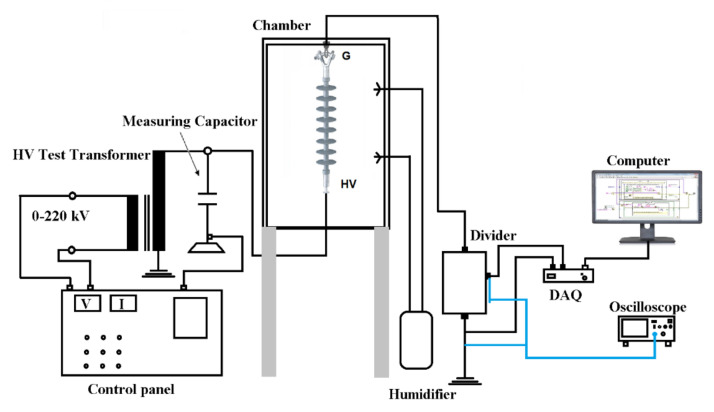
Experiment setup schematic diagram for the leakage current measurement of a polymeric insulator.

This experimental setup follows the IEC 60507 standard [29]. All studies were carried out in a 50 cm × 50 cm × 75 cm polycarbonate sheet-walled artificial test chamber. Four inlets were constructed on the test chamber wall to soak the tested insulators. The high-voltage insulator experiment setup circuit diagram is demonstrated in Figure 2. The experiment circuit consists of a high-voltage single-phase transformer (230 V/100 kV, 5 kVA, 50 Hz), a capacitor divider (100:25,000 pf), a sample test inside the chamber, a leakage current monitoring system, a steam generator with a wetting rate controller, and a voltage divider (1000:1) by resistors used to measure the leakage current.

### 2.3. Pollution and Wetting Process

Before the test was carried out, alcohol was used to remove traces of grease and dirt from all specimens. After that, the insulator samples were naturally dried for 1 day. The pollutants were deposited on the surface of the insulator using the solid layer technique [30,31,32,33]. The sodium chloride salt (NaCl) was used as Soluble Deposit Density (SDD) and the kaolin represents the Non-Soluble Deposit Density (NSDD). One liter of water was used to dissolve the sodium chloride salt and kaolin. To determine the SDD value, the electrical conductivity value of the contamination solution at room temperature was measured using a conductivity meter. The IEC 60507 standard [29] was followed to calculate SDD as in Equation (1):

(1) $$ESDD=\frac{{\left(5.7\times \sigma_{20}\right)}^{1.03}\times V}{A}$$

where *σ*_20_ is the conductivity of the pollution solution at 20 °C, *V* is the solution volume, and *A* represents the area of the insulator surface. The NSDD is calculated using Equation (2):

(2) $$NSDD=\frac{\left[(w_{s}-w_{i})\times 10^{3}\right]}{A}$$

where *w_s_* and *w_i_* are the filter paper containing pollution in dry conditions. In this work, three levels of SSD and NSDD values were estimated corresponding to light, medium, and heavy pollution, as listed in Table 2. The specimen was then contaminated and hung vertically on the artificial climate chamber handle, where the contaminated insulators were left to dry naturally for around 24 h. During the experiment, the test room pressure was constant, the same as the laboratory’s atmospheric pressure of ~99.5 kPa. The test chamber temperature was around 28 °C, which would have been the indoor temperature as in Johor Town. The rain method was used in the wetting process. Eight slots distributed regularly in the chamber wall were used to wet the tested insulators. The flow rate of the fog was controlled by the control panel located outside the HV test room. This controller was utilized to adjust the flow rate of the water and air pressure, which help to control the wetting rate of the contaminated insulators. Three levels of wetting rates were chosen: 3 l/h, 6 l/h, and 9 l/h, which were used to simulate the wetting of insulators in various climates.

The polymer insulator was tested under uniform and nonuniform pollution distribution. In non-uniform pollution, three different pollution ratios of the upper to the lower side of ESDD (F_T/B_) were chosen to be 1/5, 1/10, and 1/15. During the nonuniform application of contamination, the upper and bottom surfaces of the insulator are contaminated separately to obtain SDD_T_ and SDD_B_, while the overall SDD can be fulfilled by [5,34,35]:

(3) $$ESDD=\frac{SDD_{T}\times A_{T}+SDD_{B}\times A_{B}}{A_{T}+A_{B}}$$

where *A_T_* and *A_B_* are the areas of the top and bottom surfaces of the insulator, respectively.

### 2.4. Monitoring of Data

Referring to the experimental setup in Figure 2, the applied voltage was measured using a capacitive divider voltage and oscilloscope in the control panel. For the leakage current, the monitoring system of the LC consists of a DAQ card, PC, and oscilloscope. Because the allowable input voltage range of DAQ is ±10 V, a downscaling voltage divider (10,000:1) was used. Data were transferred from the DAQ to a PC, then saved as a CSV file, and displayed on a LabVIEW graphical user interface. For correct measurements, the oscilloscope was also used to verify the DAQ reading of data. The leakage current data stored were analyzed in the frequency domain using MATLAB software.

### 2.5. Leakage Current Features

**Figure 3:**
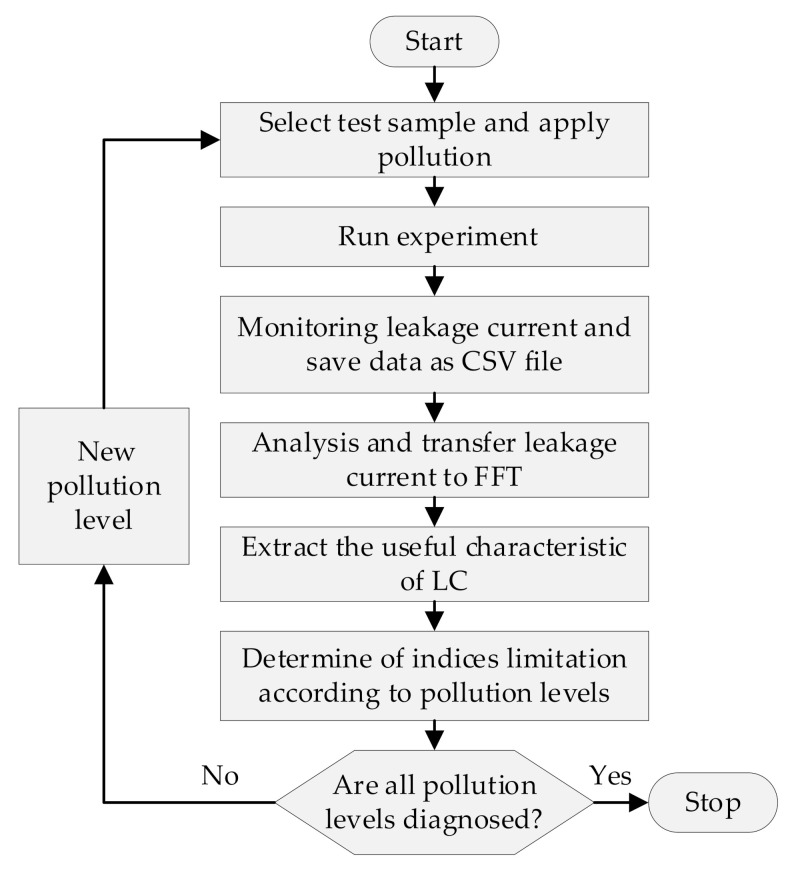
Insulator condition diagnosis using leakage current characteristics flowchart.

One way to develop new tools for diagnosing the safety state of contaminated insulators is to extract advantageous features from leakage current data. The frequency domain and time domain of the LC signal may be used to obtain these features. In this research, four indices for leakage current are recovered in both the temporal and frequency domains. Figure 3 depicts the insulator condition diagnostic procedure utilizing leakage current features.

**Figure 4:**
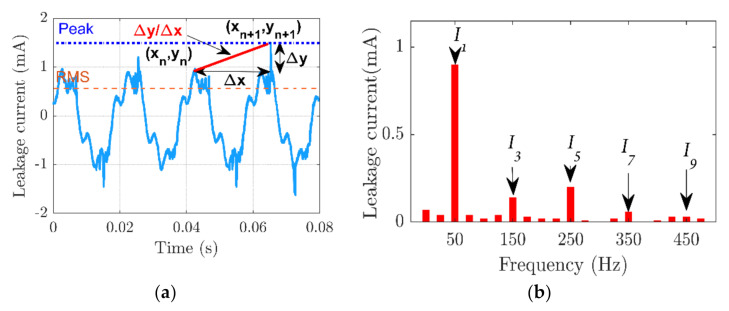
Leakage current features (**a**), time domain for extract indicators 1 and 2 (F_1_ and F_2_) (**b**), leakage current frequency domain for extract indicator 3 F_3_.

Indicators for insulator states were derived from leakage current characteristics in time and frequency domains. The slope of the line connecting two successive peaks of the leakage current signal was used to calculate the first indicator, F_1_. The second indicator, F_2_, is based on the crest factor, which was computed by dividing the peak to RMS ratio of the leakage current waveforms by two. The third, F_3_, and fourth, F_4_, indicators were determined by utilizing the odd harmonics of the leakage current under 500 Hz. The proposed indicators were expressed as follows:

(4) $$F_{1}=\frac{\sum_{n=1}^{m}\left|y_{n}-y_{n-1}\right|}{x_{n}-x_{n-1}}=\frac{\sum_{0}^{m}\left|\Delta y_{n}\right|}{\Delta x_{n}}$$

(5) $$F_{2}=\frac{I_{peak}}{I_{RMS}}$$

(6) $$F_{3}=\frac{\sum_{n}I_{n}}{I_{3}}\;n=5,7,9$$

(7) $$F_{4}=\frac{I_{5}}{I_{3}}\;$$

where ∆*y_n_* represents the difference in the leakage current between adjacent peaks at the *n^th^* point of time, ∆*x_n_* represents the time between these peaks, *I_peak_* is the peak value of the leakage current, *I_RMS_* is the root mean square of the leakage current, *I*_3_ is the third harmonic of the leakage current, *I*_5_ is the fifth harmonic of the leakage current, *I_n_* is the *n^th^* order harmonic, and *n* represents the odd-order harmonic numbers. Figure 4 depicts the leakage current characteristics in the time and frequency signals used to calculate the proposed indicators.

## 3. Results and Analysis

### 3.1. Leakage Current Findings

**Figure 5:**
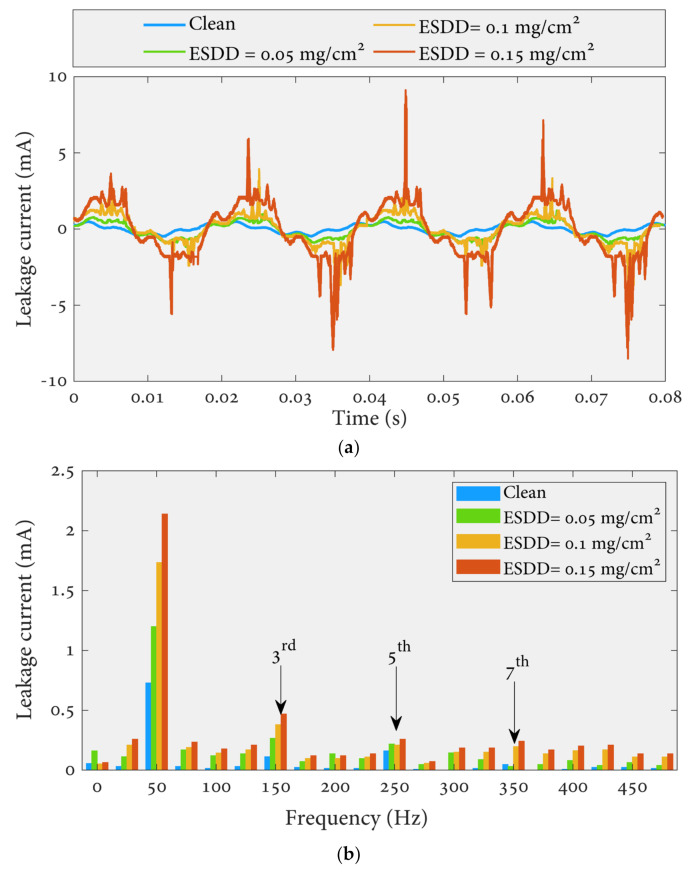
Leakage current signal under different contamination levels, 0.15 of NSDD, and 3 l/h of wetting rate: (**a**) time waveform; (**b**) FFT.

The leakage current waveform in the time domain was measured and converted to FFT using MATLAB software. Some leakage current results in time and frequency domains under different uniform pollutions, 0.15 mg/cm^2^ of NSDD, and 3 l/h of wetting rate are depicted in Figure 5. 

**Figure 6:**
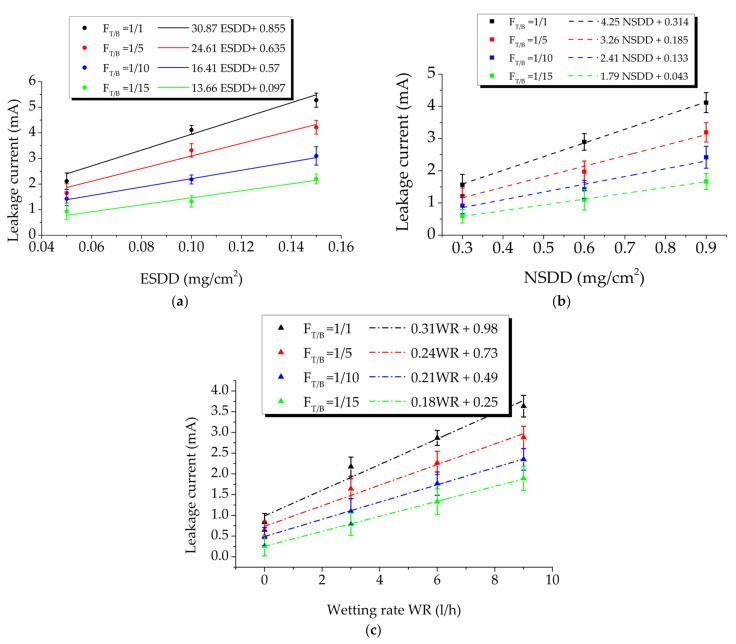
Leakage current under different values of F_T/B_. (**a**) Relationship between leakage current and ESDD when NSDD = 0.9 (mg/cm^2^) and WR = 9 (l/h); (**b**) relationship between leakage current and NSDD when ESDD = 0.1 (mg/cm^2^) and WR = 9 (l/h); (**c**) relationship between leakage current and ESDD when ESDD = 0.15 (mg/cm^2^) and NSDD = 0.9 (l/h).

Figure 5 shows a significant increase in the leakage current due to increased pollution severity under certain NSDD, wetting rate, and F_T/B_. The possible explanation for the current rising is the formation of a film due to pollution and wetness, which increases the conductivity along the surface of the insulator. As a result, an easy path was produced for the flow current in the form of positively and negatively charged ions between insulator electrodes. Under heavy pollution conditions, occasional spot-arcing was observed, especially in the presence of wetness. When the arcing was occurring, the leakage current signal became severely warped. Meanwhile, an increase in the leakage current was followed by an increase in the harmonic values. On the other hand, a significant difference in the components (3rd, 5th, 7th, and 9th) can be noticed when increasing the pollution degree on the surfaces of the insulators. The 3rd harmonic will rise to exceed the 5th, 7th, and 9th, with an apparent increase in the 7th and 9th, as reported in Figure 6. Furthermore, during arcing activity on the surface of the insulator, the third harmonic component is often considerably high [36].

Table 3 illustrates the measured leakage current components values under uniform contamination for all conditions proposed in Table 1. The experimental results showed no signs of a flashover in clean and light pollution conditions. The leakage current increased slightly as the wetting rate increased when the clean insulators were tested under different wetting conditions. This means that wetting on the insulator surface has a noticeable ability to raise the flow charges from the HV electrode to the ground electrode.

**Figure 7:**
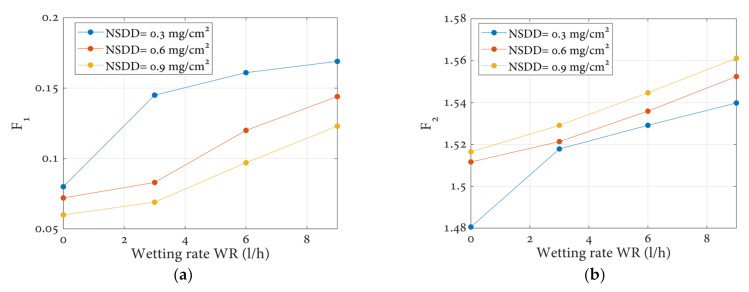

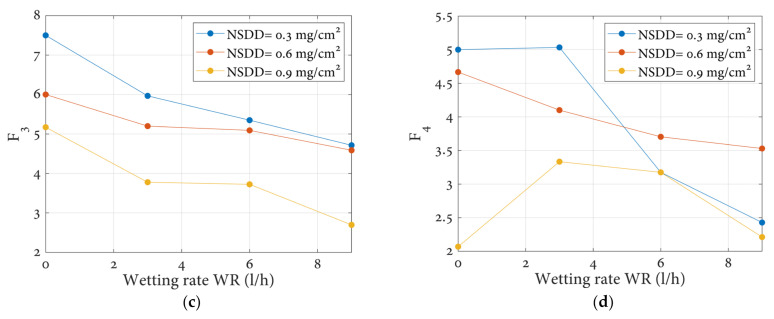
Leakage current indices of clean insulators under various wetting rates WR and NSDD: (**a**) indicator 1, F_1_; (**b**) indicator 2, F_2_; (**c**) indicator 3, F_3_; (**d**) indicator 4, F_4_.

According to Table 3, the leakage current value on a clean surface of the insulator is minimal, around 0.183 mA, and predominantly capacitive, with a phase change angle of about 90°. In a clean and dry scenario, the fifth harmonic component is always greater than the third component. Figure 6 shows the leakage current results of the polluted insulator with the change of the uneven contamination (F_T/B_), equivalate soluble deposit density (ESDD), wetting rate (WR), and non-soluble deposit density (NSDD). Overall, it can be seen from test data in Figure 6 that the leakage current is expected to grow substantially as the pollution levels (ESDD), NSDD, and wetting rate rise. Variations in leakage current amplitude increased as the ESDD, NSDD, and WR increased and Pu/Pl decreased. In dry conditions, surface conductivity is thought to be at its lowest. Accordingly, the effect of increasing the ESDD and NSDD on leakage current and its components under dry conditions was also slight. The linear fit was utilized to estimate the association between leakage current and SDD, NSSD, and the wetting rate at various (F_T/B_) values (Figure 6). The slope and intercept of linear model details are shown in Figure 7. 

### 3.2. Leakage Current Indicators Results

**Figure 8:**
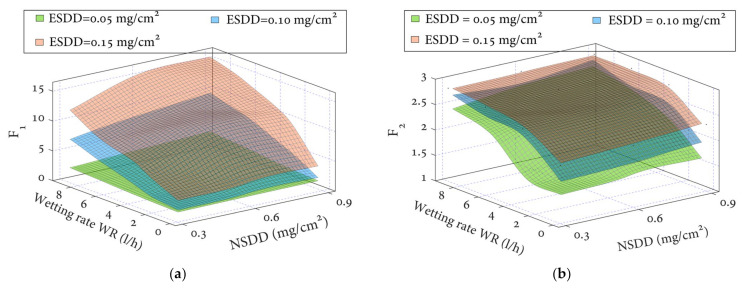

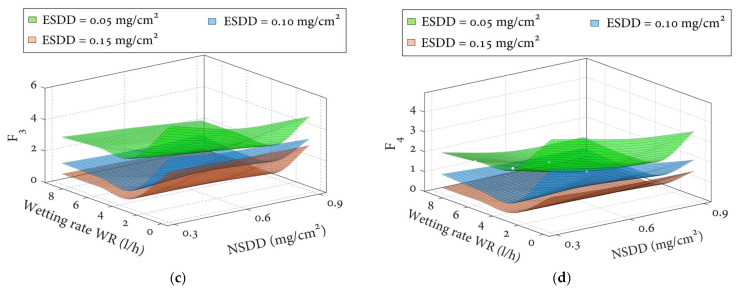
Proposed indices of uniform polluted insulators under different wetting rates, WR, and NSDD: (**a**) indicator 1, F_1_; (**b**) indicator 2, F_2_; (**c**) indicator 3, F_3_; (**d**) indicator 4, F_4_.

As previously stated, the leakage current value changes slightly as the wetting rate changes under clean conditions; similarly, the time and frequency characteristics of the leakage current will change. Figure 7 shows the leakage current indices of the clean insulator under different wetting rates. Each indicator has a special behavior when changing the wetting rate and NSDD. According to Figure 7, it can be concluded that:

(1) With an NSDD change at the same wetting rate, WR, there is a considerable influence on the presented indicators.
(2) The F_1_ and F_2_ indices increase with the increase of both the wetting rate and NSDD. It can be seen that when the WR increases from 3 l/h to 9 l/h under a certain NSDD of 0.3 mg/cm^2^, the F_1_ increases from 0.08 to 0.17 and F_2_ increases from 1.516 to 1.56.
(3) The F_3_ and F_4_ decrease with the increase of both wetting rate, WR, and NSDD. It can be observed that when the WR increases from 3 l/h to 9 l/h under a certain NSDD of 0.3 mg/cm^2^, the F_2_ decreases from 7.51 to 4.74 and F_4_ decreases from 5.09 to 2.47.
(4) At a particular wetting rate, the F_4_ is strongly impacted by harmonics in the leakage current waveform and becomes inaccurate as a monitoring indication, as seen in Figure 8d. In comparison, while the F_1_, F_2_, and F_3_ remain fairly stable under constant wetting rate regardless of harmonics in the leakage current signal, increasing the wetting rate causes an increase in the F_1_ and F_2_ and a decrease in the F_3_, making them more reliable indices for monitoring the insulator state than the F_4_.

### 3.3. Indices Trend under the Effect of Pollution Components

The leakage current indicators were studied at various levels of the pollution layer components listed in Table 2 (ESDD, NSDD, WR, and F_T/B_). Figure 8 and Table 4 demonstrate that the leakage current indices F_1_ and F_2_ of the investigated insulators rise with increasing SDD, WR, and F_T/B_. Under the same conditions, however, the insulator indices F_3_ and F_2_ decrease as the SDD increases. For instance, when the NSDD = 0.6 mg/cm^2^, WR = 6 l/h, and F_T/B_ = 1, the F_1_ value is 1.09, 6.93, and 11.46 mA whenever ESDD is 0.05, 0.10, and 0.15 mg/cm^2^, respectively. It can be seen that when the ESDD is raised from 0.05 to 0.12 and 0.2 mg/cm^2^, the F_1_ increases by 84.17% and 90.4%, respectively. Whereas, when the ESDD is 0.05, 0.12, and 0.2 mg/cm^2^, the corresponding F_3_ is 2.99, 1.24, and 0.64 mA, respectively. It can be noted that the F_1_ decreases by 58.6% and 48.83%, respectively. The results show that the indicator F_1_ changes significantly at high ESDD levels. The slopes of the F_2_, F_3_, and F_4_ values are identical across the three ESDD levels.

**Figure 9:**
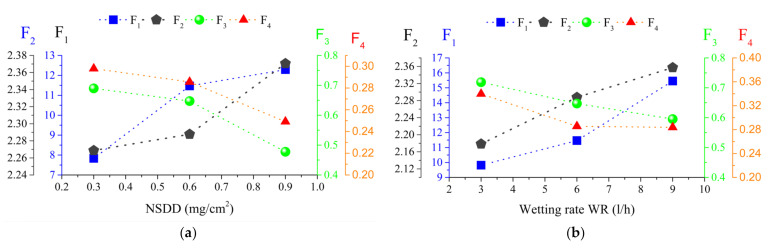

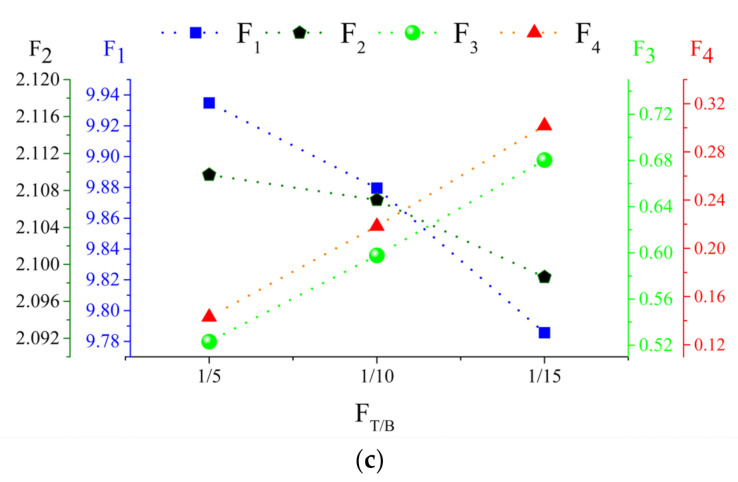
The indicator trends with (**a**) NSDD, (**b**) wetting rate (WR), and (**c**) F_T/B_.

Variations in the indices are similar to the previous situation with pollution variation. The test results in Table 4 show that for constant ESDD, WR, and F_T/B_, if the NSDD increases, the F_1_ and F_2_ will increase while the F_3_ and F_4_ will decrease. Figure 9a depicts the F_1_–F_4_ versus NSDD curves for ESDD = 0.15 mg/cm^2^, WR = 6 l/h, and F_T/B_ = 1/1 to help explain the association between NSDD and the proposed indices. The relationship between the proposed indices F1–F4 and the wetting rate, WR, for a polymer insulator under ESDD = 0.15 mg/cm^2^, NSDD = 0.9 mg/cm^2^, and F_T/B_ = 1/1 and different wetting rates, WR, is shown in Figure 9b.

It is worth noting that increasing the wetness rate WR (l/h) produces a drop in F_3_ and F_4_ and an increase in F_1_ and F_2_. For example, when ESDD = 0.2 mg/cm^2^, NSDD = 0.6 mg/cm^2^, and F_T/B_ = 1/1, F_1_ rises by 14.6% and 16.8%, respectively, when the WR increases from 3 to 6 and 9 l/h. In contrast, when the WR is increased from 3 to 6 and 9 l/h, the F_3_ decreases by 9.3 percent and 15.7 percent, respectively.

Figure 9c demonstrates the connection between the suggested indices F_1_–F_4_ and F_T/B_ for polluted polymer insulators with ESDD = 0.15 mg/cm^2^, NSDD = 0.9 mg/cm^2^, and WR = 9 l/h. It can be seen that increasing the F_T/B_ produces a drop in the F_1_ and F_2_ while increasing the F_3_ and F_4_. This suggests that insulation under uniform pollution levels is more severe than under non-uniform pollution situations.

### 3.4. Insulator Condition Based on Indices Ranges Based on the Test Data

**Figure 10:**
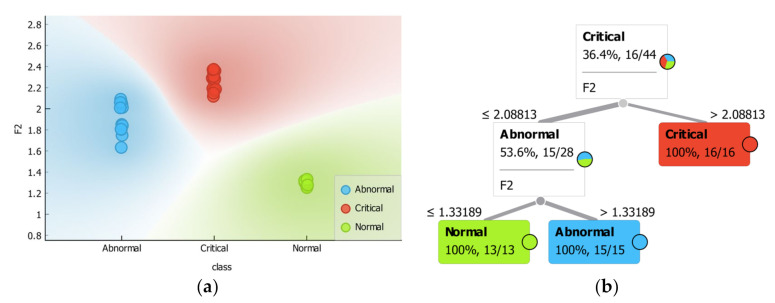
(**a**) Boundary detection of indicator 2 F_2_ (as an example); (**b**) classification tree for F_2_ indicator to estimate the insulator condition.

In this part, the insulator condition was characterized based on the range of the indicators recovered experimentally that correlate to the levels of ESDD, NSDD, WR, and FT/B. The experimental results show that the F_1_ and F_2_ rise when increasing the ESDD, NSDD, and WR and decreasing F_T/B_. In contrast, the indices F_3_ and F_4_ drop when the ESDD, NSDD, and WR increase and F_T/B_ decreases. The proposed indicator ranges were estimated using classification tree methods. As an example, Figure 10 depicts the decision boundaries and trained decision tree for indicator F_2_. Table 5 shows the insulator condition prediction based on the indicator’s values extracted experimentally. Accordingly, Table 4 and Table 5 demonstrate that:

(1) In the clean and light pollution scenarios, the suggested indicator values were observed in the normal range, with a WR less than 3.8 l/h and an NSDD less than 0.45 mg/cm^2^. The probability of a discharge occurring in this scenario is almost non-existent.
(2) The insulator was in abnormal condition under light pollution (0.05 mg/cm^2^) with a high WR (9 l/h) and moderate and heavy levels of NSDD (0.6 and 0.9 mg/cm^2^) for all contaminated distribution FT/B except the 1/5 level, according to the ranges of indicators in Table 5. Furthermore, in the presence of medium contamination (0.12 mg/cm^2^), medium WR (6 l/h), NSDD (0.6 mg/cm^2^), and FT/B (1/1 to 1/5), the insulator under investigation showed an abnormal state. Except in situations of high wetting, in which the probability of flashover increases, the probability of a discharge happening in these conditions is limited.

**Table 5:** Insulator condition dependent on experimentally determined indices values.

Indicator	Normal Range	Abnormal Range	Critical Range
F_1_	<1.36	>1.5 and <5.5	>5.5
F_2_	<1.33	>1.33 and <2.1	>2.1
F_3_	>3.4	>1.5 and <3.4	<1.5
F_4_	>2.2	>1.2 and <2.2	<1.2

(1) The critical condition of the insulator under testing was discovered in two states: first, under moderate pollution levels for WR (9 l/h), NSDD (0.9 mg/cm^2^), and all pollution distribution categories; and second, under heavy pollution conditions for WR, NSDD, and all pollution distribution scenarios. In these conditions, the probability of a discharge occurring is significant, especially under severe wetting and heavy NSDD.
(2) The pre-flashover metric values of the indices indicate that these metrics can be employed to detect the flashover occurrence for polluted insulators during service.

## 4. Determination of Indices Performance

**Figure 11:**
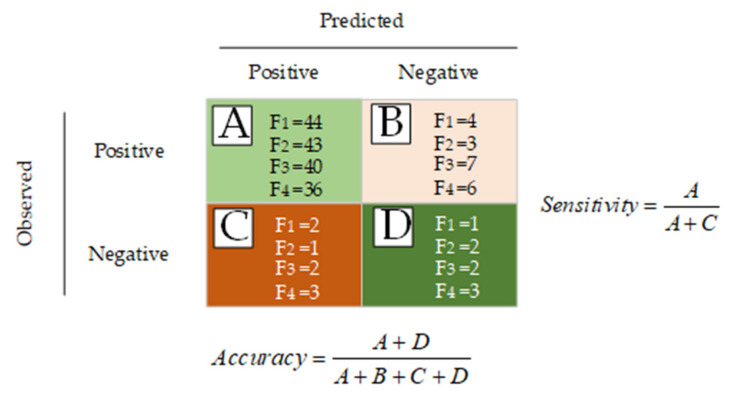
Confusion matrix for determining indices’ sensitivity, specificity, and accuracy.

The performance of the suggested indicators to accurately estimate the insulator state from the 196 test observations was investigated. The confusion matrix illustrated in Figure 11 was used to compute the sensitivity and accuracy of these indices. The values of the confusion matrix were determined based on the capability of the insulator indicator to predict the correct condition. The selection of test results and recommended indices are specified as follows:

A: The results of the test and the indicator prediction are both positive (correct).
B: The test result is positive, but the indicator prediction is negative.
C: The test result is negative, but the indicator prediction is positive.
D: The results of the test and the indicator prediction are both negative (incorrect).

The insulator condition was successfully reflected in the majority of tests (170 out of 196). In contrast, 26 of the test findings were negative. The erroneous test findings might be related to a lack of adequate implementation of contaminants on the insulator surface, a measurement equipment mistake, or other factors.

**Figure 12:**
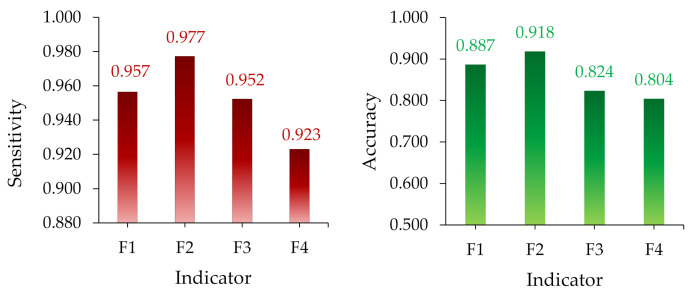
The value of the indices’ sensitivity, specificity, and accuracy for evaluating the insulator condition.

The diversity in the number of projected outcomes may help determine which indicators are the best, with the indices with the greatest number of correct predicted outcomes being the best. In other words, the indices with the greatest number of correctly predicted results will be the most accurate. Based on the indices’ prediction results presented in the figure above, indicator F_1_ (44) had the highest number of correct predictions, followed by F_2_ (43), F_3_ (40), and F_4_ (36). The indicator measures (sensitivity and accuracy) were calculated as shown in Figure 12. Figure 12 shows that indicator F_2_ has the highest sensitivity and accuracy, followed by F_1_, F_3_, and F_4_, respectively.

## 5. Conclusions

Based on laboratory investigations conducted according to the IEC 60507 standard for polymer insulators under pollution, the leakage current has been measured. The leakage current value shows a positive slope when the ESDD, WR, and NSDD on the surface of insulators increase. However, the slope will be negative when F_T/B_ increases. The LC value is mostly affected by the pollution level, ESDD, and wetness rate, WR. Four indicators have been extracted from leakage current signals at different pollution levels. The results show that the suggested indicators are able to evaluate the insulator condition effectively. The indicators F_1_ and F_2_ indicate that the insulators will be in critical condition if their values exceed 5.5 mA and 2.1, respectively. If the F_3_ and F_4_ values are less than 1.5 and 1.2, respectively, the insulator will also be in the critical state. The possibility of flashover occurrence becomes higher when the insulator condition becomes critical. Moreover, the results show that all indicators presented in this study are useful for determining the condition of polluted insulators. However, based on the confusion matrix analysis, the indicator F_2_ performs better compared to other indicators. The current analysis can therefore be utilized to devise a reasonable criterion in identifying changes in the leakage current characteristics. When the measured leakage current reaches a particular threshold value, the associated change can be better identified. The suggested approach can be implemented online and a prototype device for monitoring the insulator condition can be developed in the future to verify the practicality of the suggested approach.

## Figures and Tables

**Table 1:** Insulator parameters.

Parameter	Symbol	Length (cm)
Leakage distance	L_t_	89
Height	H	52.5
Diameter	D	9.8
Core diameter	Dc	2.4
Arc distance	Ha	32.9

**Table 2:** Pollution layer components.

Parameters	Values			
Pollution level	Clean (C)	Light (L)	Medium (M)	Heavy (H)
ESDD (mg/cm^2^)	0.0	0.05	0.10	0.15
NSDD (mg/cm^2^)	0.0	0.3	0.6	0.9
Wetting rate (l/h)	0.0	3	6	9

**Table 3:** Leakage current components under uniform conditions.

ESDD	NSDD	WR	I_1_	I_3_	I_5_	I_7_	I_9_
0.00	0	0	0.1483	0.0004	0.0016	0.0000	0.0016
	0.3	0	0.1621	0.0005	0.0024	0.0008	0.0004
		3	0.3566	0.0024	0.0122	0.0016	0.0006
		6	0.5835	0.0032	0.0103	0.0065	0.0006
		9	0.7293	0.0057	0.0138	0.0081	0.0049
	0.6	0	0.1783	0.0024	0.0113	0.0016	0.0016
		3	0.5429	0.0081	0.0332	0.0041	0.0049
		6	0.7050	0.0088	0.0324	0.0065	0.0057
		9	0.7861	0.0138	0.0486	0.0081	0.0065
	0.9	0	0.3323	0.0047	0.0097	0.0073	0.0073
		3	0.5997	0.0146	0.0486	0.0041	0.0024
		6	0.7374	0.0162	0.0515	0.0049	0.0041
		9	0.8509	0.0267	0.0592	0.0057	0.0073
0.05	0.3	0	0.4230	0.0073	0.0348	0.0073	0.0032
		3	0.7293	0.1013	0.2593	0.0486	0.0243
		6	0.9643	0.1102	0.2431	0.0673	0.0324
		9	1.2723	0.1459	0.2917	0.0746	0.0624
	0.6	0	0.5916	0.0113	0.0454	0.0065	0.0057
		3	0.9968	0.1151	0.1872	0.0689	0.0535
		6	1.2075	0.1353	0.1864	0.1135	0.0567
		9	1.4911	0.1451	0.1872	0.1216	0.0648
	0.9	0	0.7374	0.0146	0.0527	0.0105	0.0081
		3	1.3209	0.1540	0.2042	0.0827	0.0454
		6	1.6532	0.1783	0.1945	0.1135	0.0729
		9	2.1151	0.2115	0.2593	0.0972	0.0891
0.10	0.3	0	0.5154	0.0178	0.0583	0.0089	0.0065
		3	1.1994	0.2674	0.2188	0.0324	0.0648
		6	1.3209	0.3323	0.2593	0.0502	0.0972
		9	1.5640	0.3728	0.3323	0.0770	0.0648
	0.6	0	0.6078	0.0259	0.0681	0.0105	0.0170
		3	1.3938	0.2836	0.2512	0.0843	0.0729
		6	1.5883	0.4295	0.2998	0.0891	0.0794
		9	1.8963	0.4619	0.3323	0.1053	0.0891
	0.9	0	0.7618	0.0332	0.0713	0.0251	0.0186
		3	1.6045	0.4538	0.2917	0.1053	0.1297
		6	1.6532	0.5835	0.3323	0.1216	0.1053
		9	2.1151	0.5024	0.3404	0.1378	0.1459
0.15	0.3	0	0.6823	0.0429	0.0778	0.0365	0.0259
		3	2.1394	0.7780	0.2269	0.1702	0.1216
		6	2.3825	0.8023	0.1864	0.2107	0.0891
		9	2.9579	0.8809	0.1864	0.2188	0.1053
	0.6	0	0.8347	0.0502	0.0810	0.0511	0.0259
		3	2.3744	0.7699	0.2269	0.1702	0.0891
		6	2.6661	0.8833	0.2188	0.1864	0.0972
		9	3.2334	1.0535	0.2593	0.1945	0.0972
	0.9	0	0.8671	0.0527	0.0827	0.0389	0.0405
		3	2.8687	1.1280	0.2431	0.0972	0.1216
		6	4.2464	1.3128	0.2836	0.1702	0.0972
		9	5.2755	1.4506	0.2188	0.1053	0.1378

**Table 4:** Leakage current indicators of polymer insulators under variation non-uniform pollution F_T/B_, wetting rate (WR), and NSDD.

F_T/B_			1/5				1/10				1/15			
ESDD mg/cm^2^	NSDD mg/cm^2^	W_t_ l/h	F_1_	F_2_	F_3_	F_4_	F_1_	F_2_	F_3_	F_4_	F_1_	F_2_	F_3_	F_4_
0.05	0.3	0	0.11	1.18	7.92	6.14	0.08	0.81	5.45	4.23	0.05	0.47	8.32	6.26
		3	0.30	1.26	3.89	2.68	0.21	0.87	2.68	1.84	0.14	0.51	4.09	2.73
		6	0.64	1.29	3.62	2.28	0.44	0.89	2.49	1.57	0.30	0.52	3.80	2.33
		9	0.85	1.30	3.46	2.08	0.58	0.89	2.38	1.43	0.39	0.52	3.64	2.12
	0.6	0	0.25	1.31	7.78	6.60	0.17	0.90	5.35	4.54	0.12	0.53	8.18	6.73
		3	0.57	1.28	3.21	1.97	0.39	0.88	2.21	1.36	0.26	0.51	3.37	2.01
		6	0.89	1.29	3.06	1.92	0.61	0.89	2.11	1.32	0.41	0.52	3.22	1.96
		9	2.56	1.30	3.04	1.97	1.76	0.89	2.09	1.36	1.19	0.52	3.19	2.01
	0.9	0	0.43	1.32	7.12	5.31	0.30	0.91	4.90	3.65	0.20	0.53	7.48	5.42
		3	0.72	1.29	2.48	1.55	0.50	0.89	1.71	1.07	0.33	0.52	2.61	1.58
		6	2.51	1.30	2.50	1.26	1.73	0.89	1.72	0.87	1.16	0.52	2.63	1.29
		9	4.66	1.31	2.41	1.26	3.21	0.90	1.66	0.87	2.16	0.53	2.53	1.29
0.10	0.3	0	0.52	1.33	6.07	4.85	0.36	0.92	4.18	3.34	0.24	0.53	6.38	4.95
		3	1.87	1.27	1.48	1.08	1.29	0.87	1.02	0.74	0.87	0.51	1.56	1.10
		6	3.89	1.31	1.51	0.96	2.68	0.90	1.04	0.66	1.80	0.53	1.59	0.98
		9	6.70	1.84	1.68	1.17	3.23	1.27	1.16	0.81	2.18	0.74	1.77	1.19
	0.6	0	0.65	2.00	5.76	4.31	0.45	1.38	3.96	2.97	0.30	0.80	6.05	4.40
		3	4.66	1.33	1.64	1.02	3.21	0.92	1.13	0.70	2.16	0.53	1.72	1.04
		6	5.59	1.80	1.49	0.95	3.85	1.24	1.03	0.65	2.59	0.72	1.57	0.97
		9	7.11	2.00	1.49	0.95	4.89	1.38	1.03	0.65	3.30	0.80	1.57	0.97
	0.9	0	0.66	2.14	4.74	2.96	0.45	1.47	3.26	2.04	0.31	0.86	4.98	3.02
		3	4.27	1.34	1.32	0.81	2.94	0.92	0.91	0.56	1.98	0.54	1.39	0.83
		6	6.08	1.83	1.32	0.79	4.18	1.26	0.91	0.54	2.82	0.73	1.39	0.81
		9	8.57	2.06	1.36	0.79	5.90	1.42	0.94	0.54	3.97	0.83	1.43	0.81
0.15	0.3	0	0.85	2.19	3.90	2.18	0.58	1.51	2.68	1.50	0.39	0.88	4.10	2.22
		3	4.87	1.46	0.77	0.21	3.35	1.00	0.53	0.14	2.26	0.59	0.81	0.21
		6	6.32	1.97	0.72	0.27	4.35	1.36	0.50	0.19	2.93	0.79	0.76	0.28
		9	9.33	2.08	0.68	0.27	5.04	1.43	0.47	0.19	3.40	0.83	0.71	0.28
	0.6	0	1.05	2.24	3.74	1.94	0.72	1.54	2.57	1.34	0.49	0.90	3.93	1.98
		3	7.92	1.50	0.80	0.35	5.45	1.03	0.55	0.24	3.67	0.60	0.84	0.36
		6	9.25	2.03	0.71	0.30	6.37	1.40	0.49	0.21	4.29	0.81	0.75	0.31
		9	12.46	2.05	0.61	0.37	8.58	1.41	0.42	0.25	5.78	0.82	0.64	0.38
	0.9	0	0.52	2.18	3.65	1.88	0.36	1.50	2.51	1.29	0.24	0.87	3.84	1.92
		3	8.58	1.60	0.64	0.34	5.91	1.10	0.44	0.23	3.98	0.64	0.67	0.35
		6	9.91	2.06	0.76	0.39	6.82	1.42	0.52	0.27	4.60	0.83	0.80	0.40
		9	9.93	2.11	0.52	0.14	6.83	1.45	0.36	0.10	4.61	0.85	0.55	0.14

## Data Availability

Not applicable.
